# Supplementary material for: Anti-Inflammatory Potential of Cow, Donkey and Goat Milk Extracellular Vesicles as Revealed by Metabolomic Profile
Source: Nutrients. 2020 Sep 23;12(10):2908. doi: 10.3390/nu12102908 (PMC7598260; doi:10.3390/nu12102908)
Supplement: Supplementary file 1 [file nutrients-12-02908-s001.zip › Supplementary/FigS10.pdf]

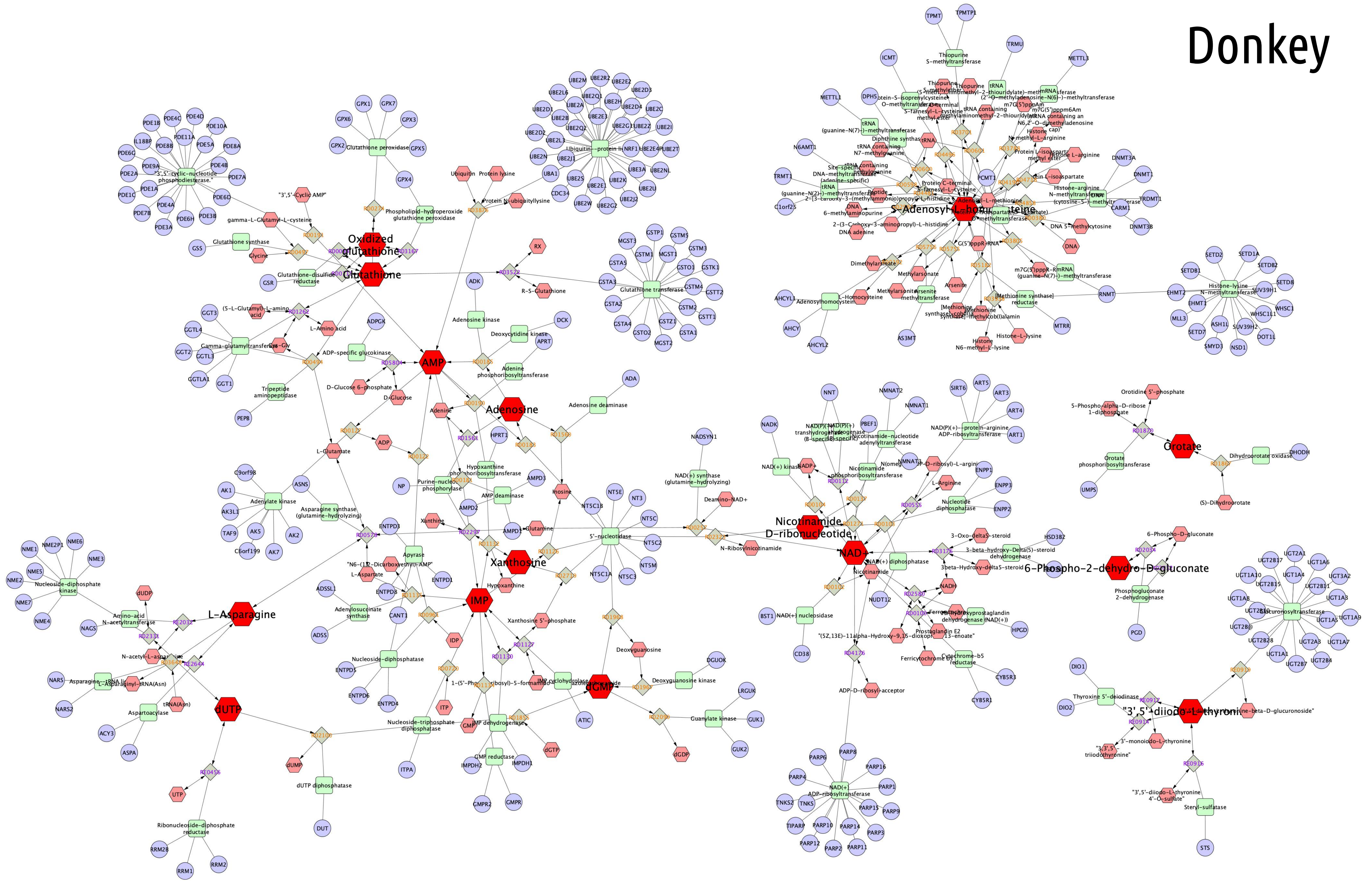

Figure S10. Donkey total compound-reaction-enzyme-gene. Network of metabolites highly expressed in MEVs.
