# Supplementary material for: Anti-Inflammatory Potential of Cow, Donkey and Goat Milk Extracellular Vesicles as Revealed by Metabolomic Profile
Source: Nutrients. 2020 Sep 23;12(10):2908. doi: 10.3390/nu12102908 (PMC7598260; doi:10.3390/nu12102908)
Supplement: Supplementary file 1 [file nutrients-12-02908-s001.zip › Supplementary/FigS4.pdf]

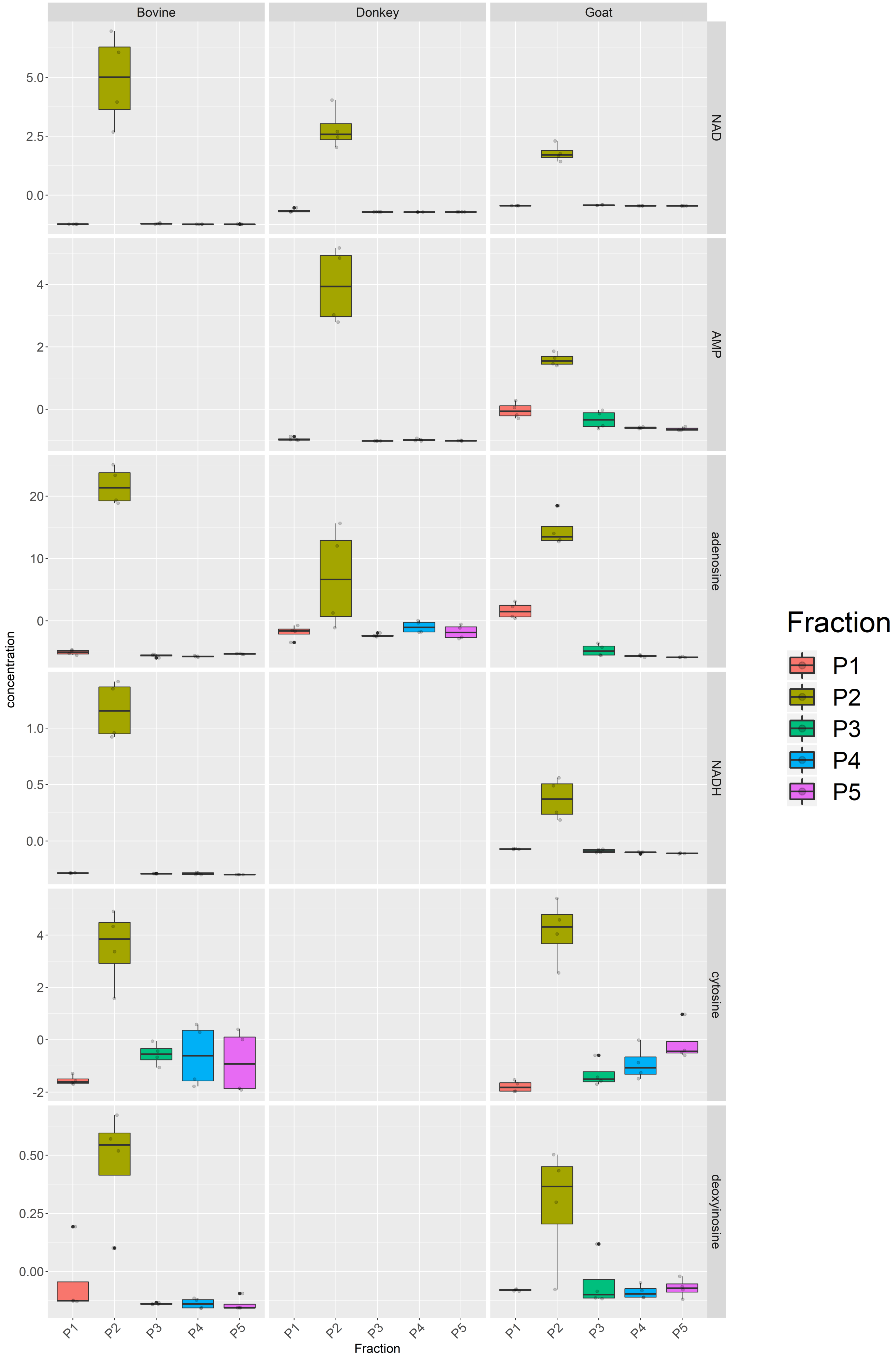

Figure S4. Comparison of metabolites enriched in P2 fractions among Bovine, Donkey and Goat milk. For each fraction, four samples were analyzed. The normalized concentration is reported for each metabolite in the P1, P2, P3, P4, P5 pools. The distribution of the samples is represented by a boxplot. The solid line represents the median, the box reports the interquartile range and the whiskers reports the minimum and maximum value of the distribution. Each sample is represented by a grey point, outliers are reported using a black point.

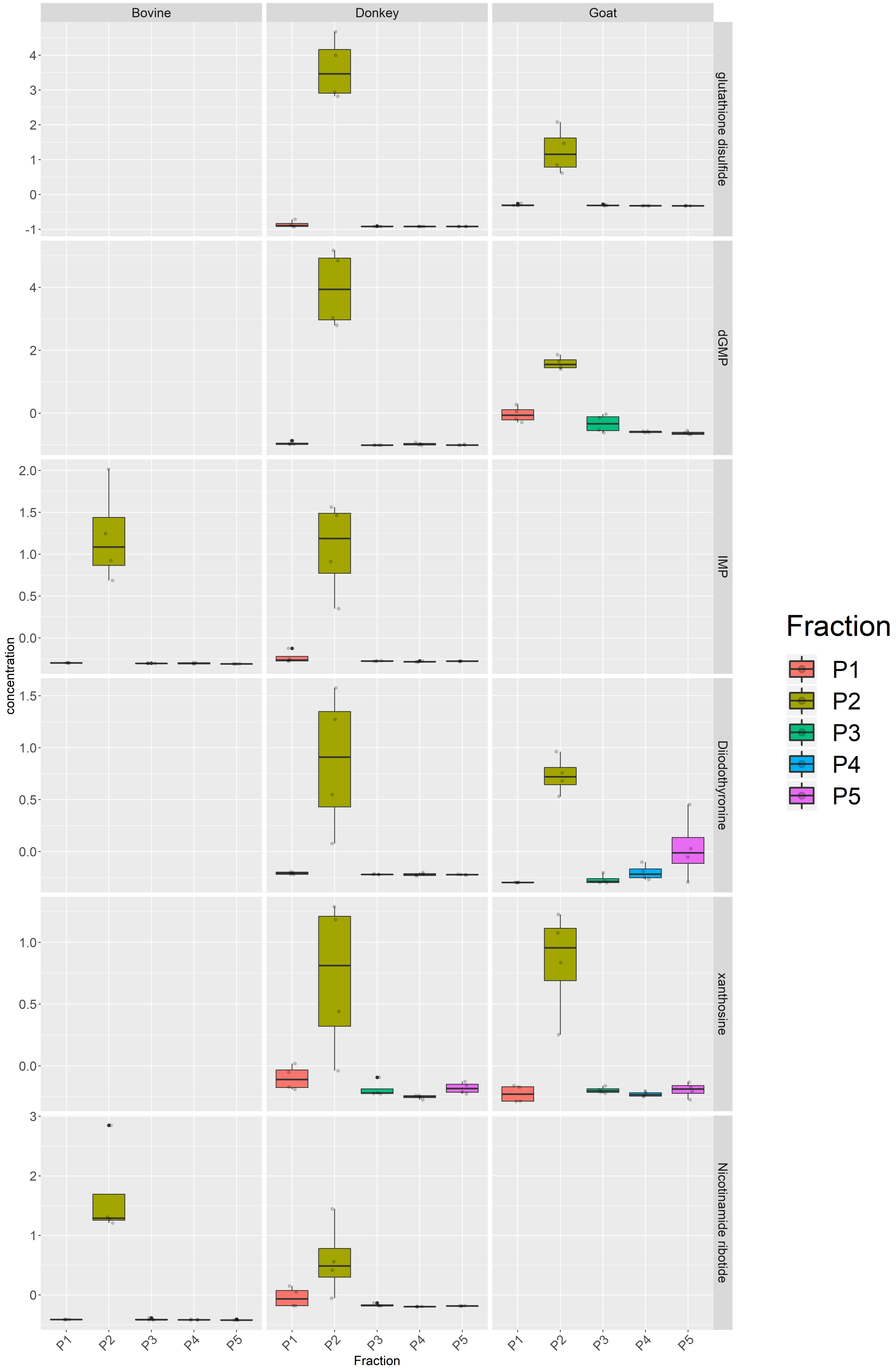
Bovine
Donkey
Goat
glutathione disulfide
dGMP
IMP
Diiodothyronine
xanthosine
Nicotinamide ribotide
concentration
Fraction
P1
P2
P3
P4
P5
Fraction
P1
P2
P3
P4
P5

Figure S4
