# Supplementary material for: Anti-Inflammatory Potential of Cow, Donkey and Goat Milk Extracellular Vesicles as Revealed by Metabolomic Profile
Source: Nutrients. 2020 Sep 23;12(10):2908. doi: 10.3390/nu12102908 (PMC7598260; doi:10.3390/nu12102908)
Supplement: Supplementary file 1 [file nutrients-12-02908-s001.zip › Supplementary/FigS5.pdf]

Vitamin B3 (nicotinate and nicotinamide) metabolism

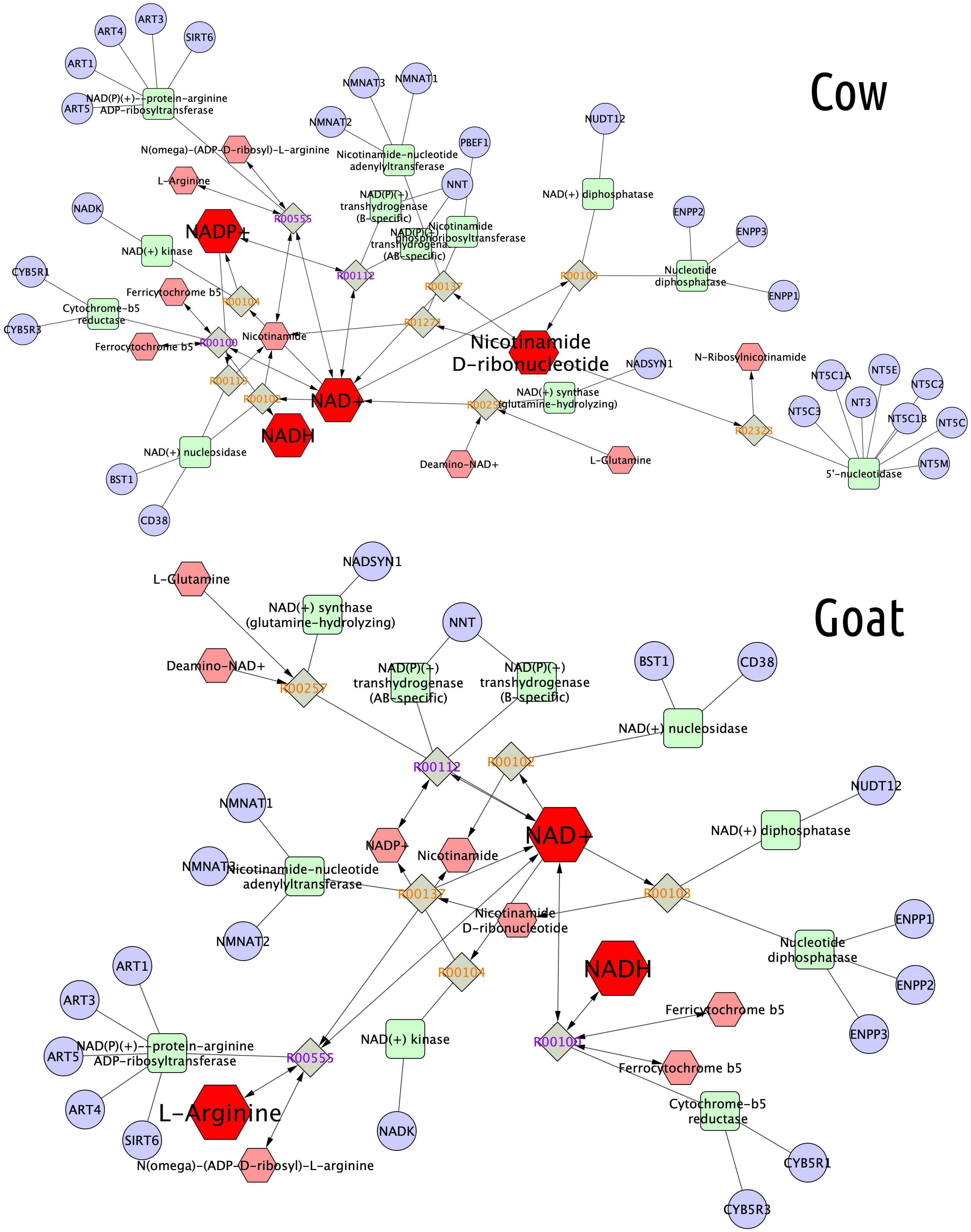

Figure S5. Visual representation generated by MetScape analyses of the CREGN for common pathways among the three species, involving one or more MEV exclusive metabolites. Red hexagons symbolize these input MEVs metabolites, arrows indicate their connections with other compounds (pink hexagons), genes (purple circles), enzymes (green squares) and reactions (beige rhombuses).

Vitamin B3 (nicotinate and nicotinamide) metabolism

Donkey

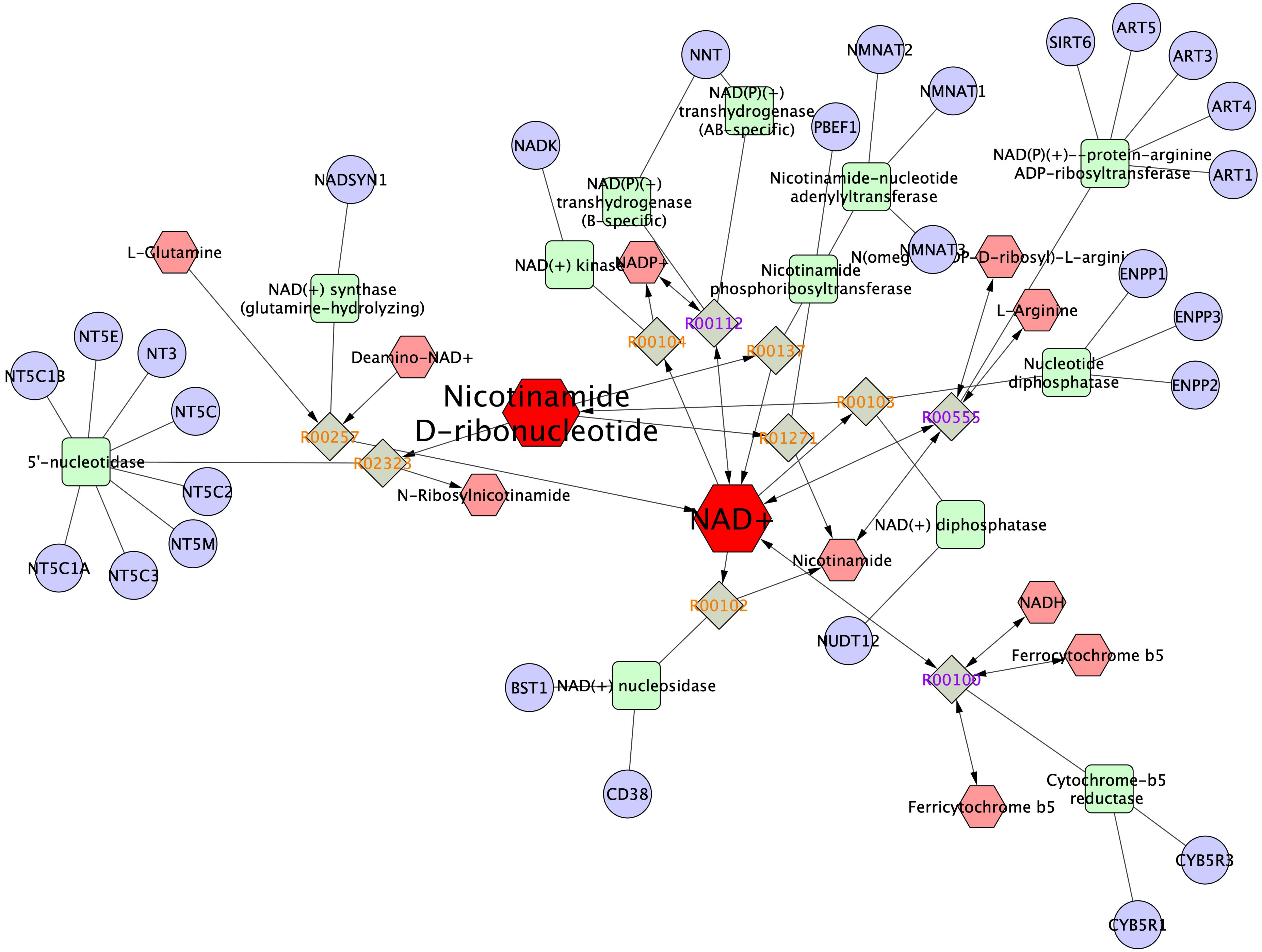

Figure S5

Purine metabolism

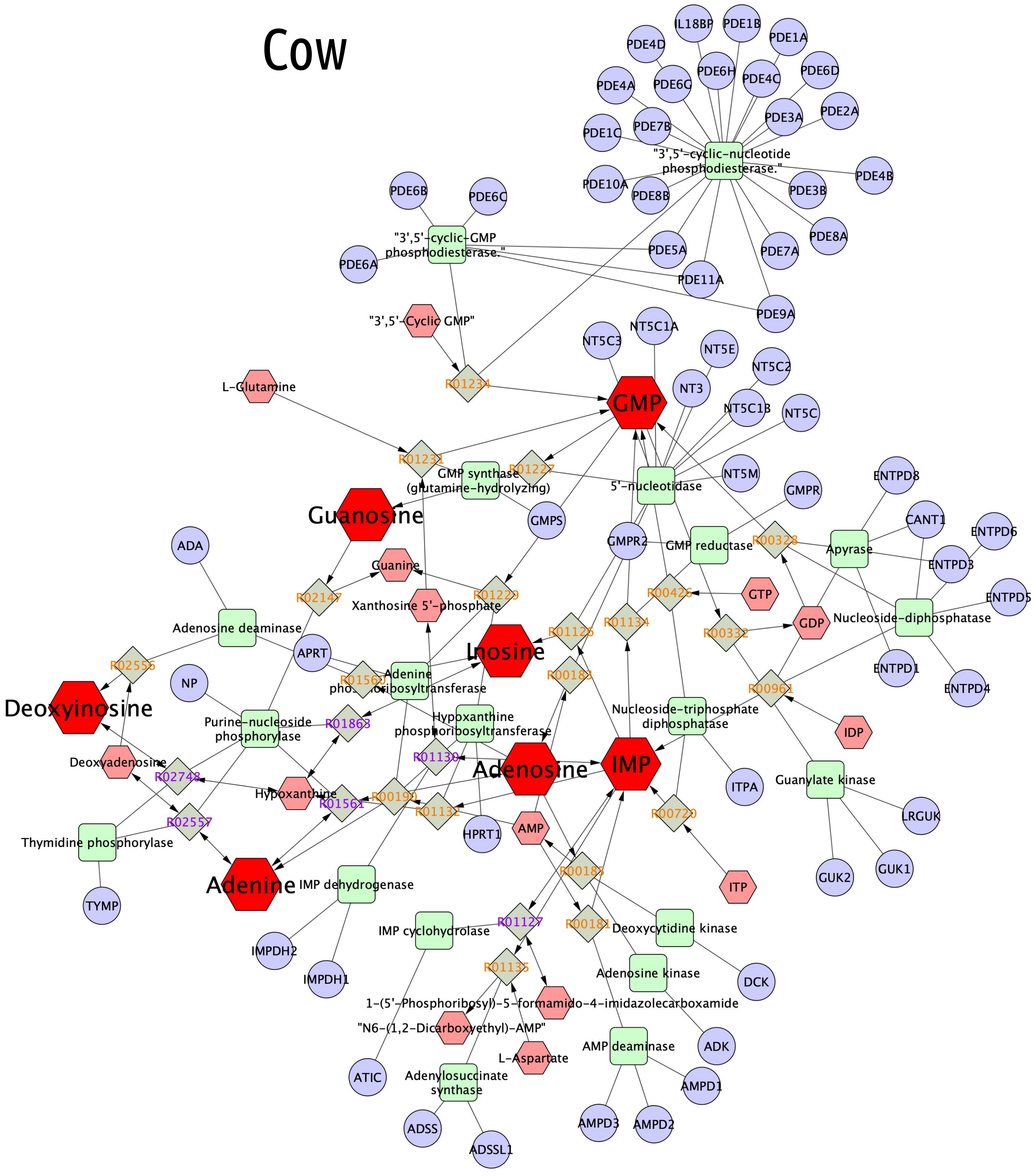

Figure S5

Purine metabolism

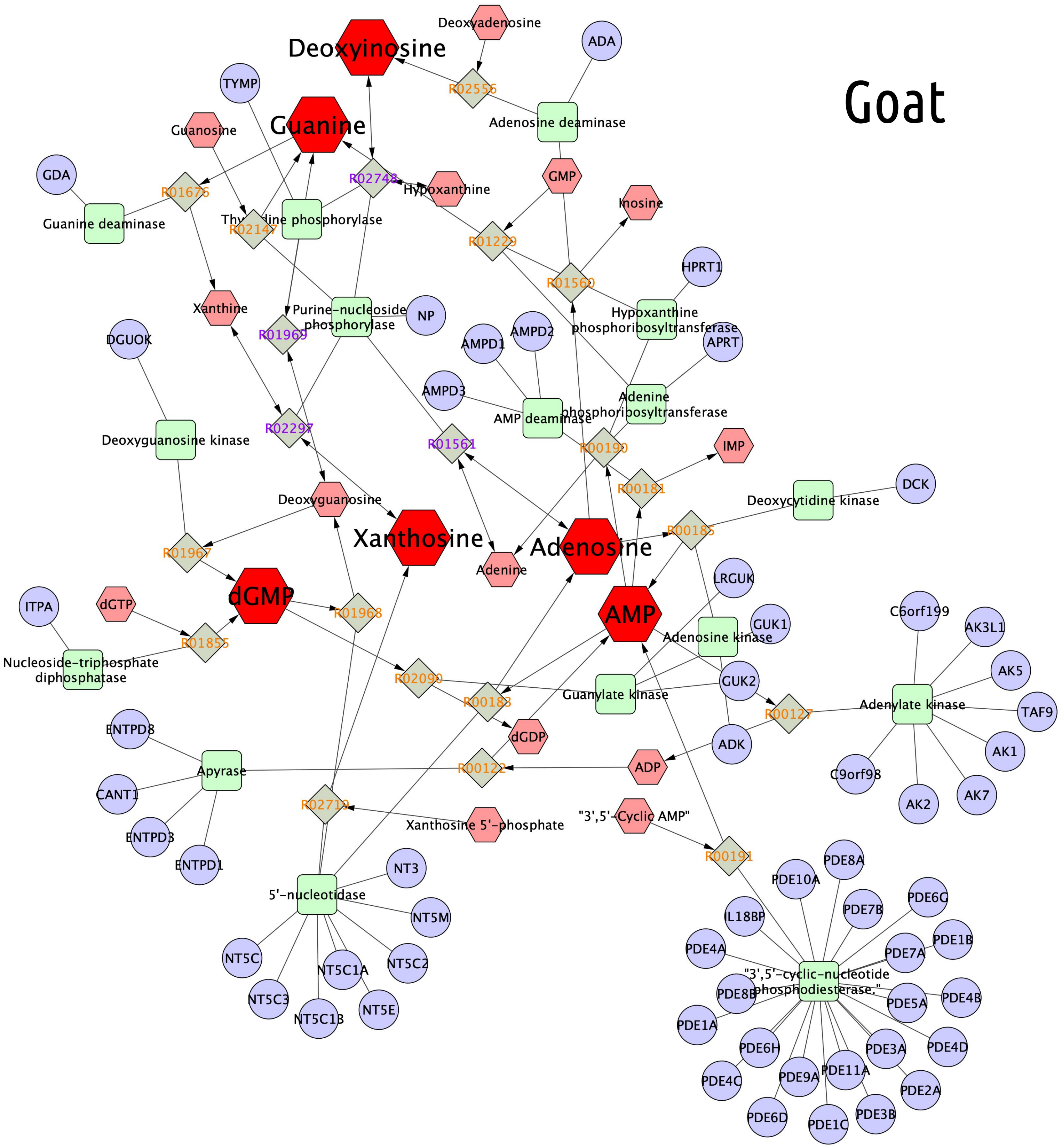

Figure S5

Purine metabolism

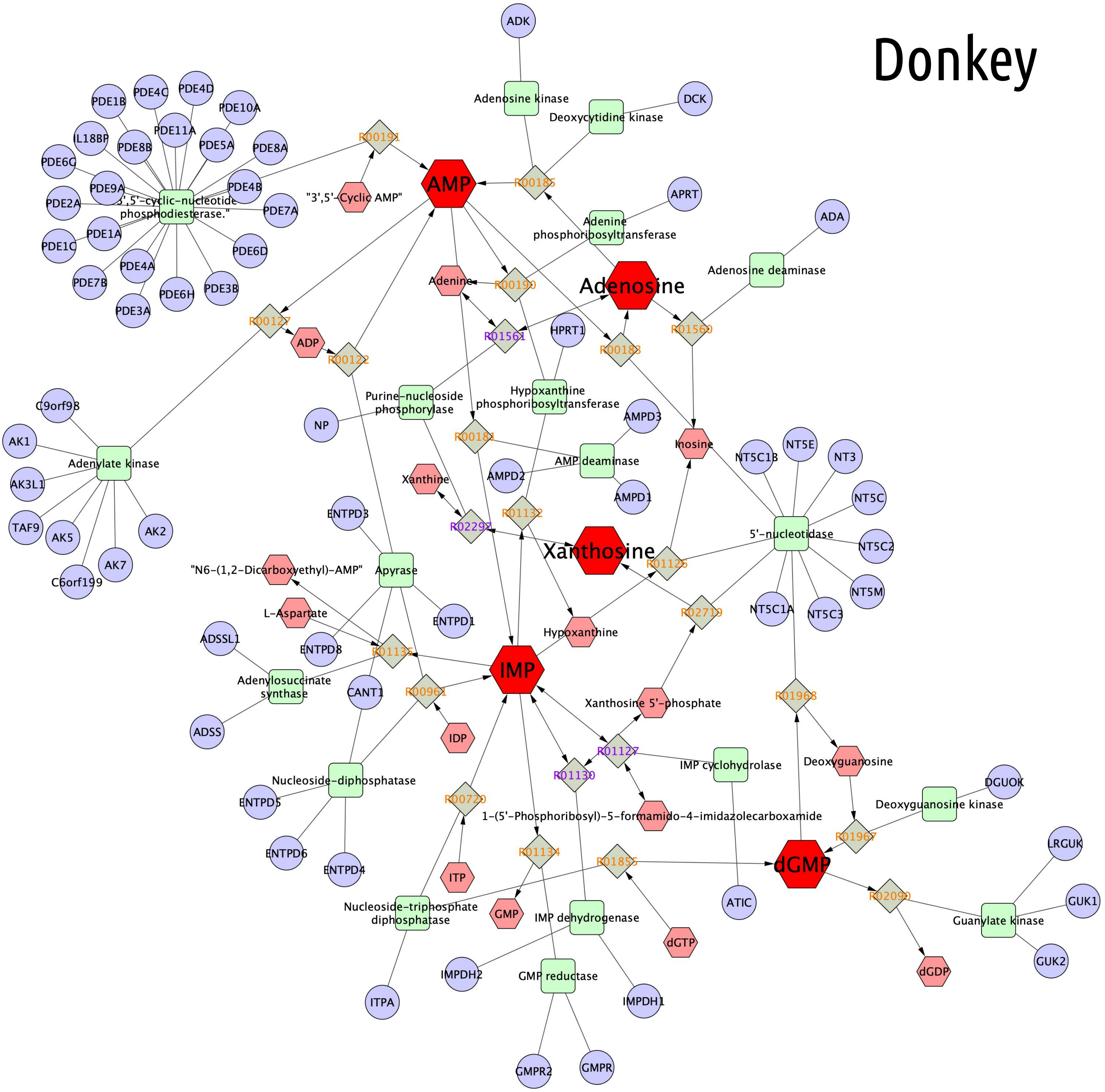

Figure S5

Pyrimidine metabolism

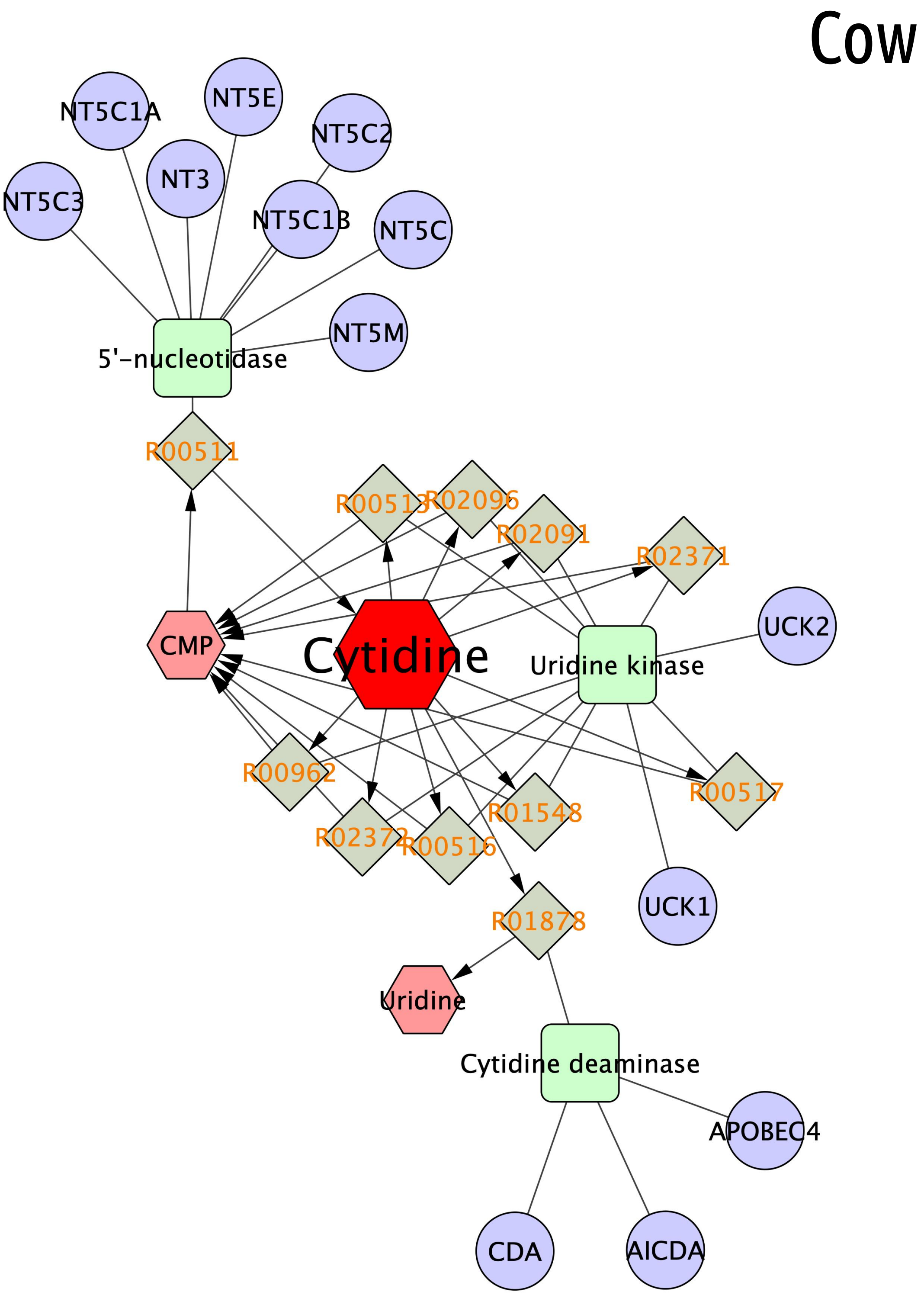

Figure S5

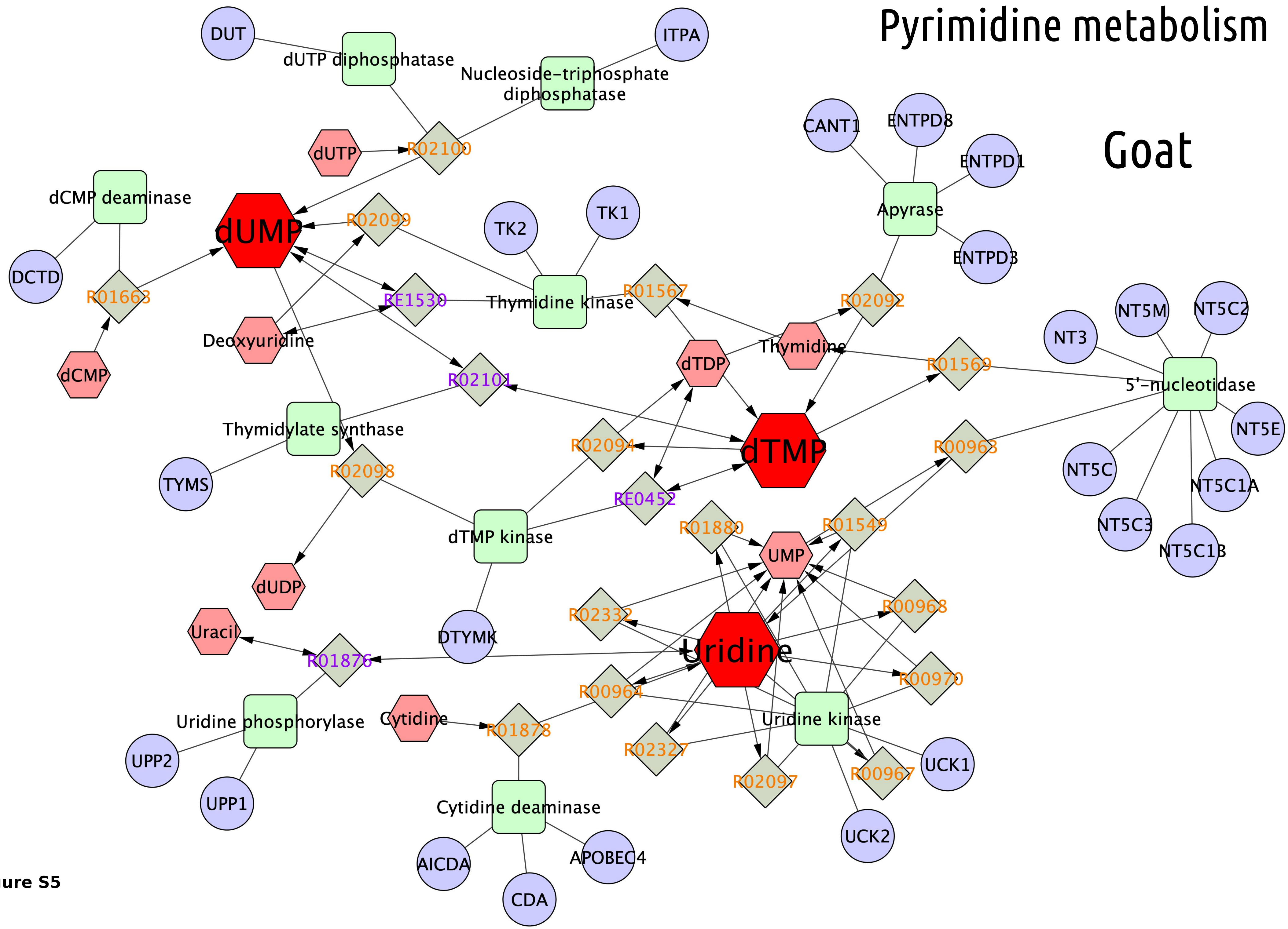

Pyrimidine metabolism
Goat
DUT
ITPA
dUTP diphosphatase
Nucleoside-triphosphate diphosphatase
dUTP
R02100
dCMP deaminase
dUMP
R02099
TK2
TK1
CANT1
ENTPD8
ENTPD1
Apyrase
ENTPD3
DCTD
R01663
RE1530
Thymidine kinase
R01567
R02092
Deoxyuridine
dCMP
dTDP
Thymidine
NT5M
NT5C2
NT3
R02101
R01569
5'-nucleotidase
Thymidylate synthase
R02094
dTMP
R00963
NT5E
TYMS
R02098
RE0452
NT5C
NT5C1A
dTMP kinase
R01880
R01549
NT5C3
NT5C1B
UMP
dUDP
R02332
R00968
Uracil
DTYMK
Uridine
R01876
R00970
R00964
Uridine phosphorylase
Cytidine
R01878
Uridine kinase
R02327
UPP2
R02097
R00967
UCK1
UPP1
Cytidine deaminase
UCK2
AICDA
APOBEC4
CDA

Figure S5

Pyrimidine metabolism

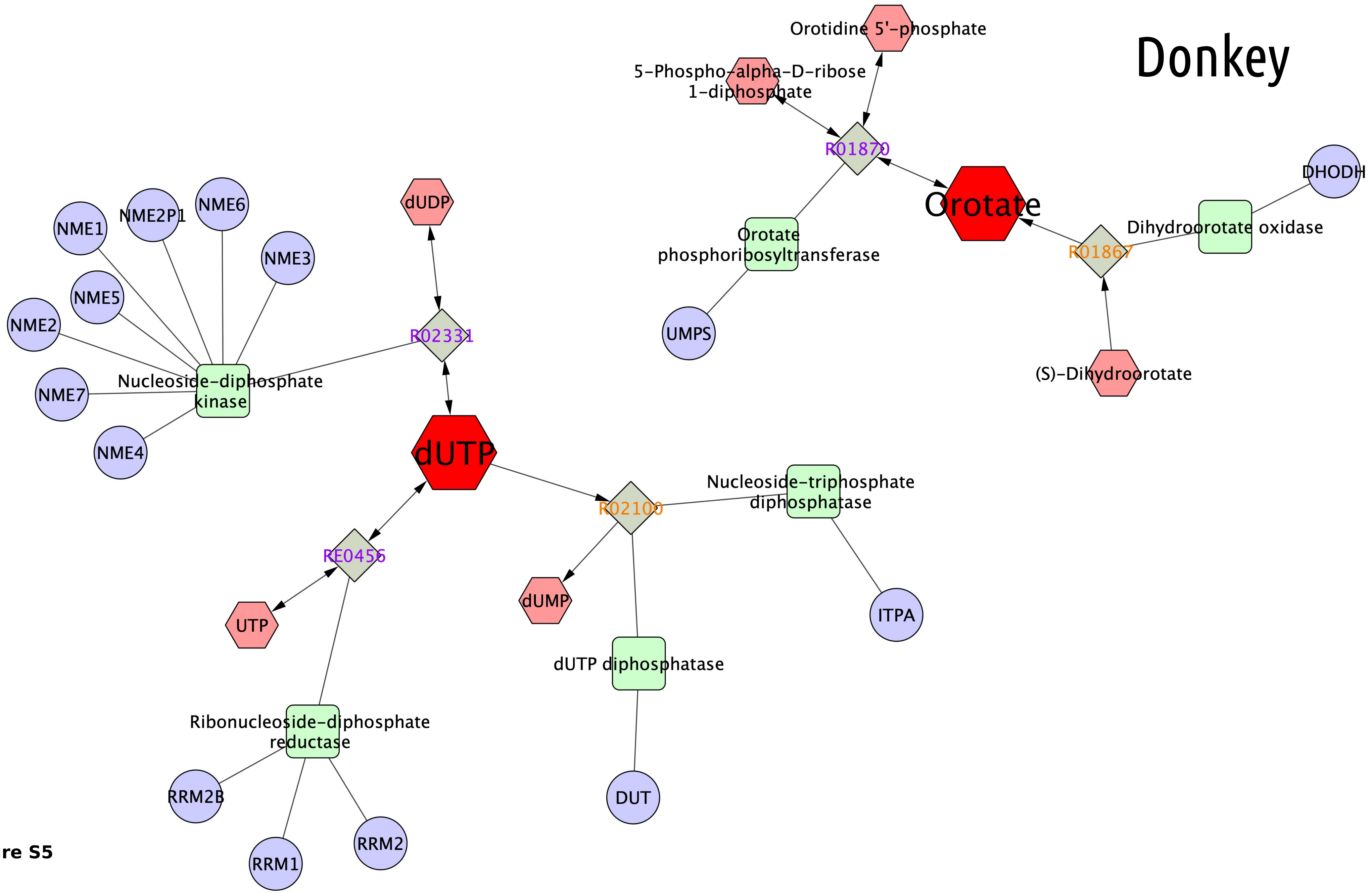

Figure S5
