# Supplementary material for: Anti-Inflammatory Potential of Cow, Donkey and Goat Milk Extracellular Vesicles as Revealed by Metabolomic Profile
Source: Nutrients. 2020 Sep 23;12(10):2908. doi: 10.3390/nu12102908 (PMC7598260; doi:10.3390/nu12102908)
Supplement: Supplementary file 1 [file nutrients-12-02908-s001.zip › Supplementary/FigS6.pdf]

Tyrosine metabolism

Donkey

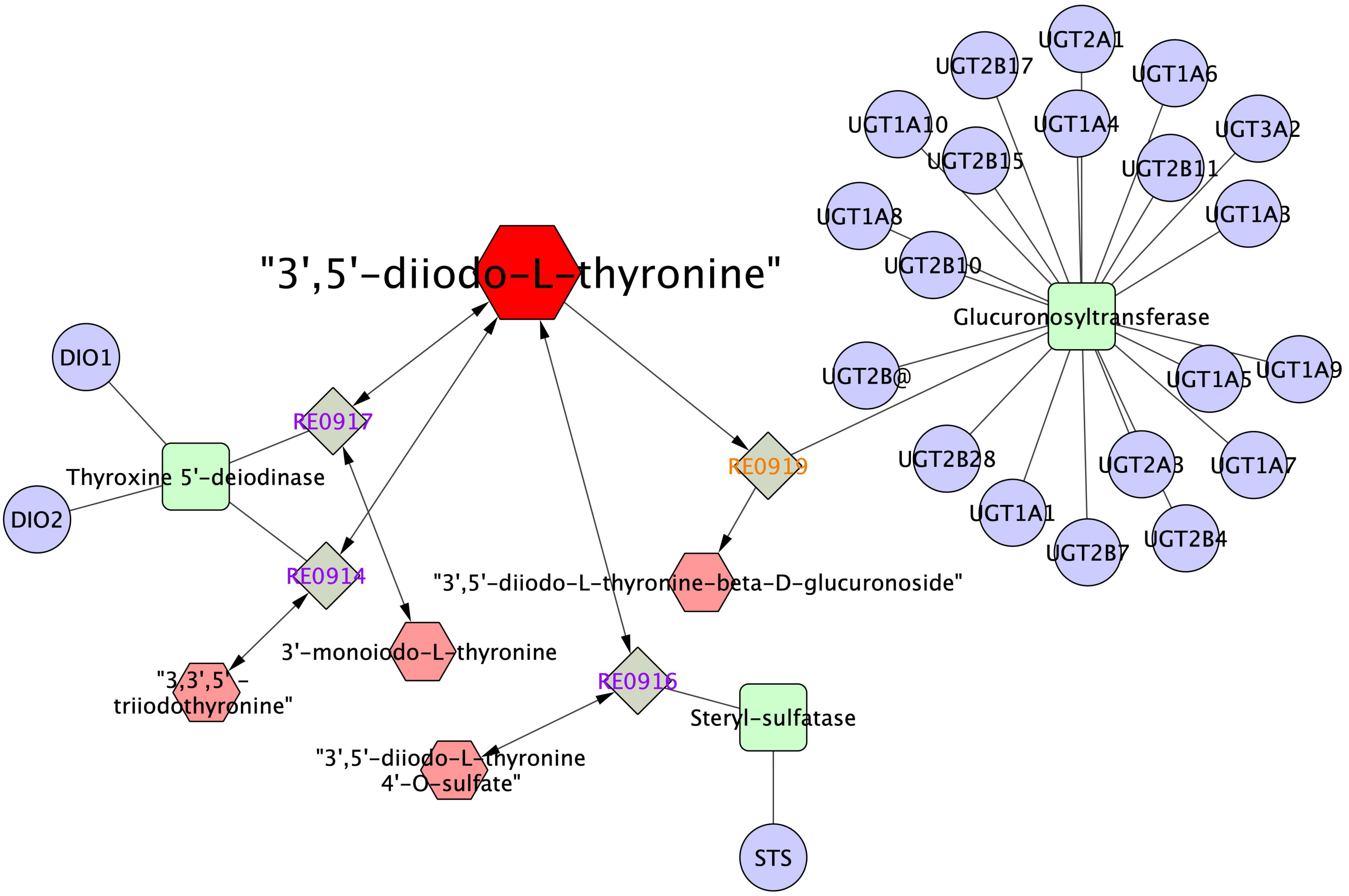

Figure S6. Visual representation generated by MetScape analyses of the CREGN for common pathways among two species, involving one or more MEV exclusive metabolites.

Tyrosine metabolism

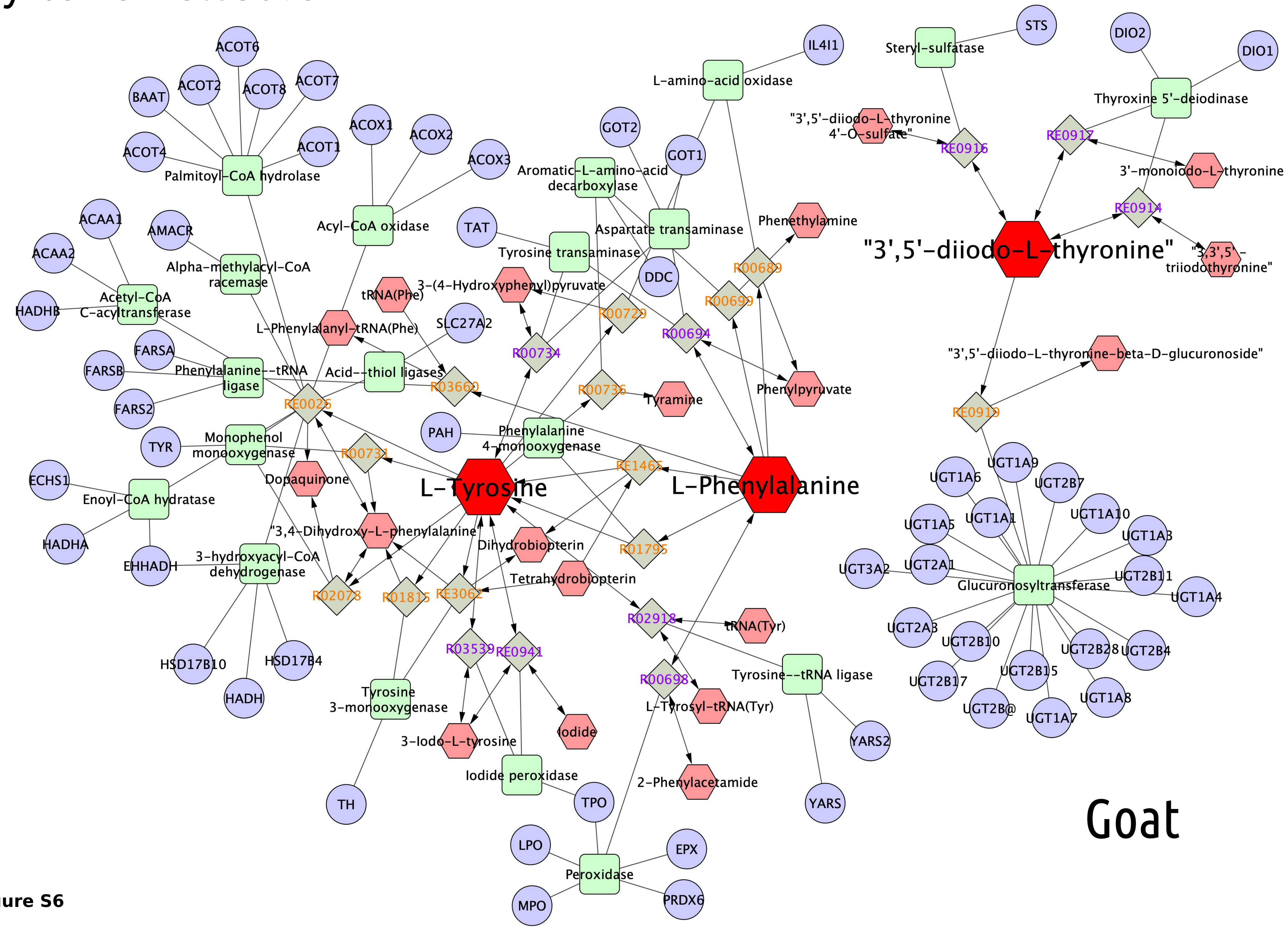

Figure S6

Methionine and cysteine metabolism

Goat

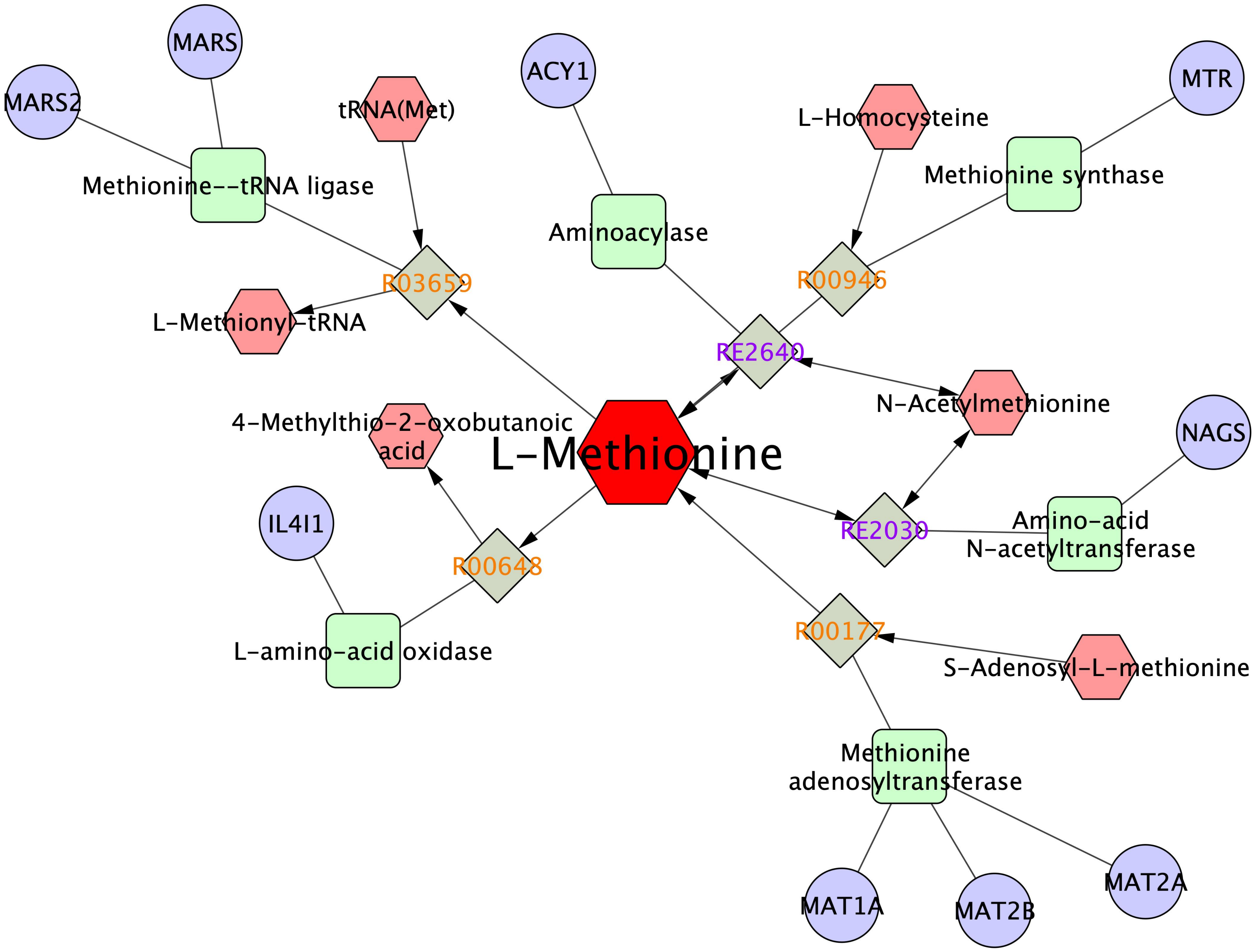

Figure S6

Methionine and cysteine metabolism

Donkey

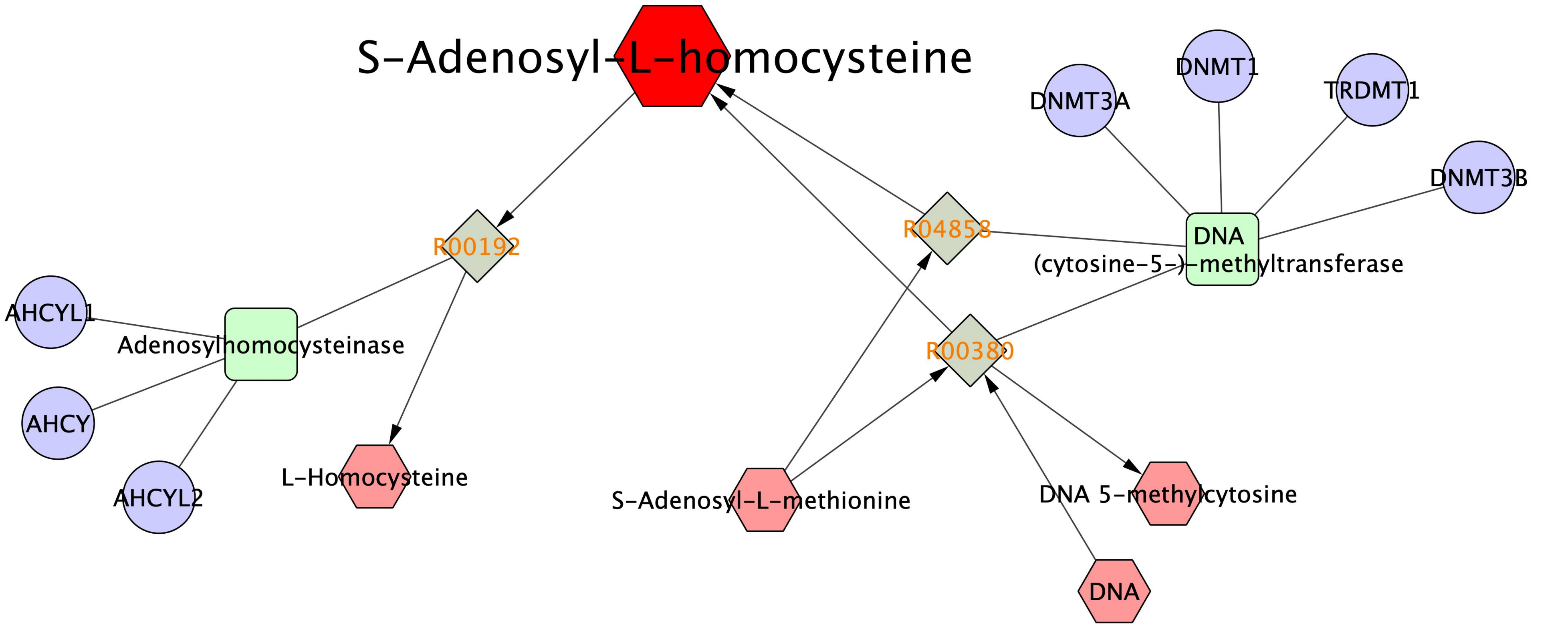

Figure S6
