# Supplementary material for: Anti-Inflammatory Potential of Cow, Donkey and Goat Milk Extracellular Vesicles as Revealed by Metabolomic Profile
Source: Nutrients. 2020 Sep 23;12(10):2908. doi: 10.3390/nu12102908 (PMC7598260; doi:10.3390/nu12102908)
Supplement: Supplementary file 1 [file nutrients-12-02908-s001.zip › Supplementary/FigS7.pdf]

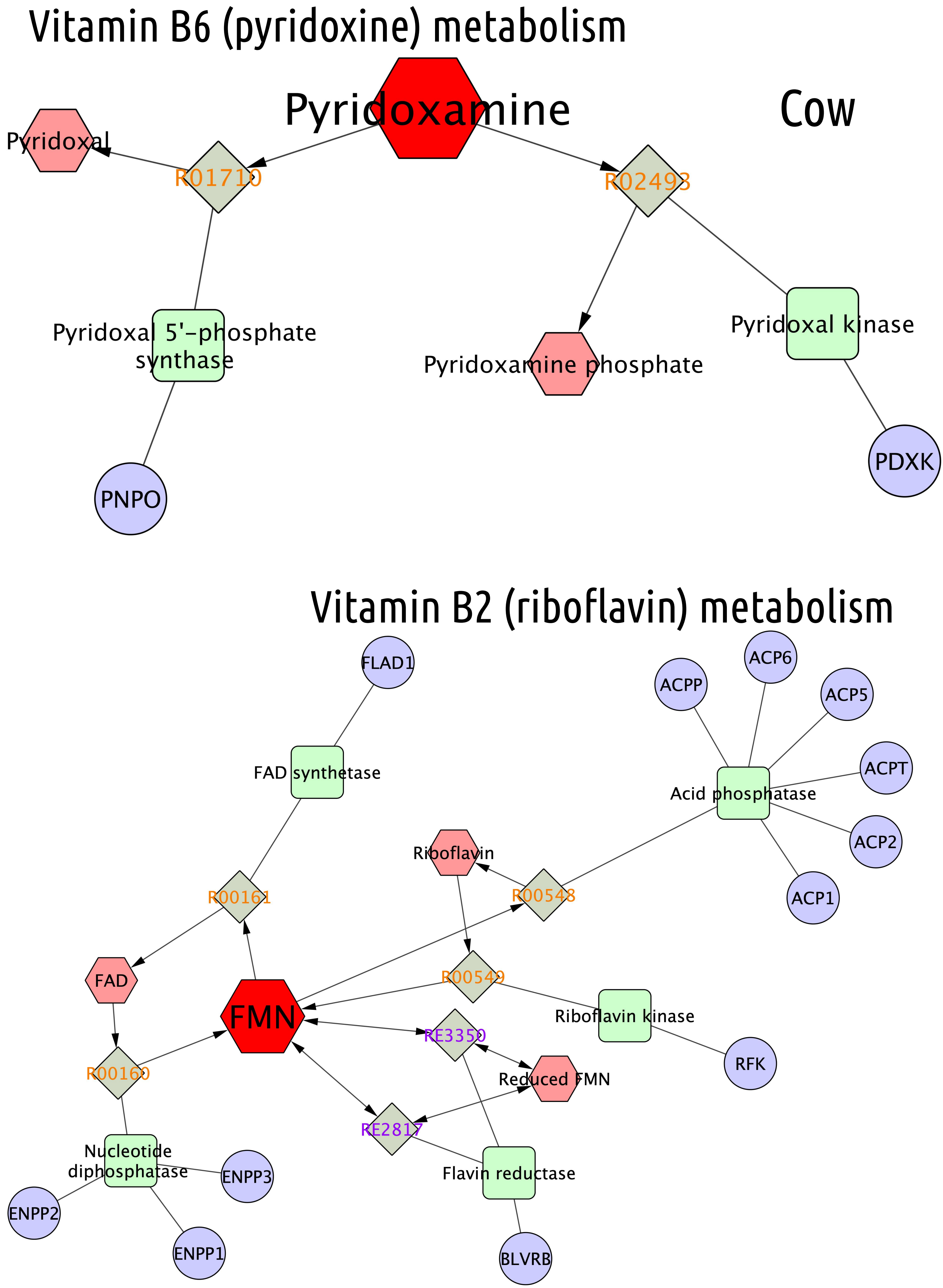

Figure S7. Peculiar pathway of a specific species emerged with the MetScape analysis (CREGN).

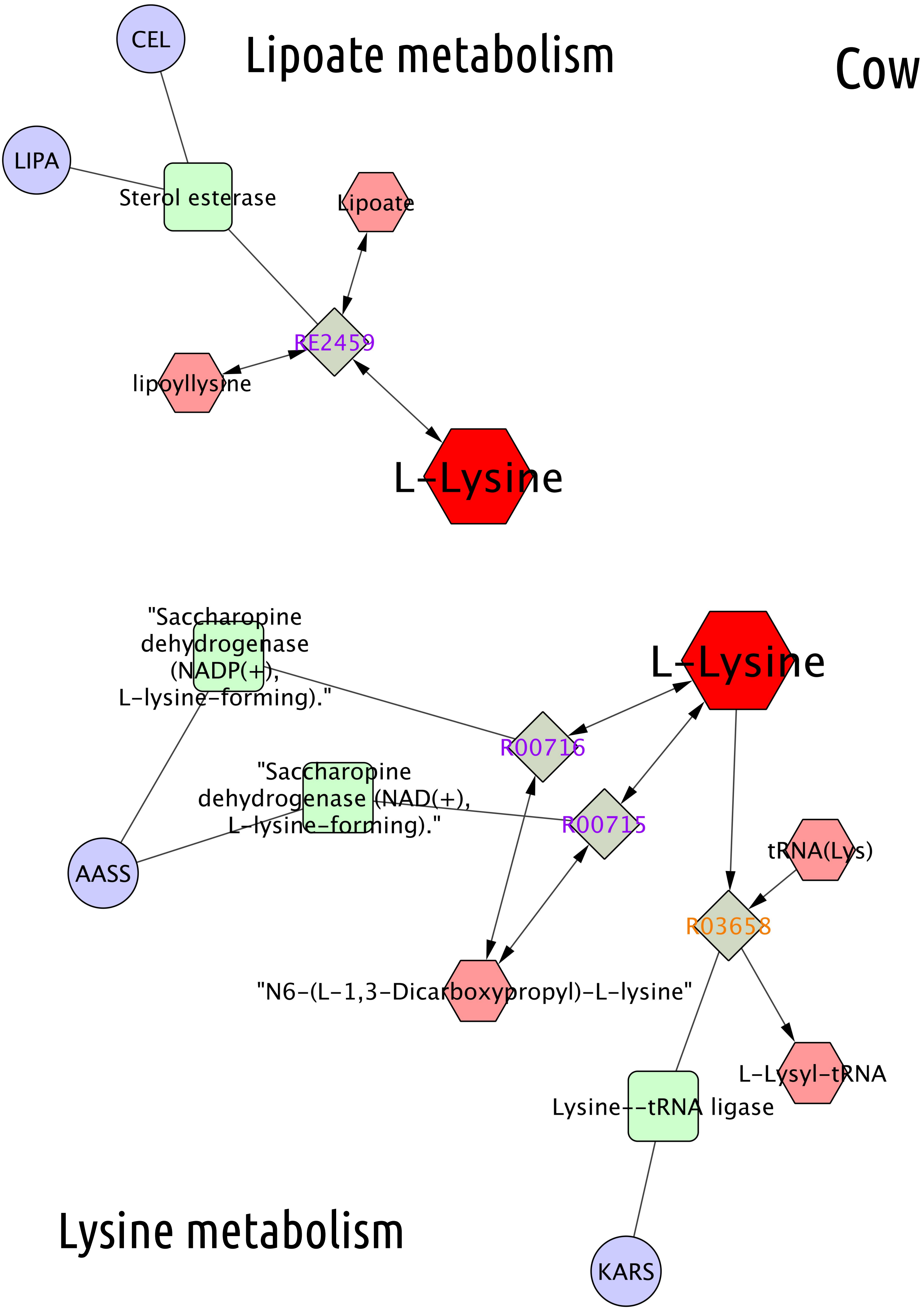
Lipoate metabolism
Cow
CEL
LIPA
Sterol esterase
Lipoate
RE2459
lipoyllysine
L-Lysine
"Saccharopine dehydrogenase (NADP(+), L-lysine-forming)."
L-Lysine
R00716
"Saccharopine dehydrogenase (NAD(+), L-lysine-forming)."
R00715
AASS
tRNA(Lys)
R03658
"N6-(L-1,3-Dicarboxypropyl)-L-lysine"
L-Lysyl-tRNA
Lysine--tRNA ligase
Lysine metabolism
KARS

Figure S7

Vitamin H (biotin) metabolism

Cow

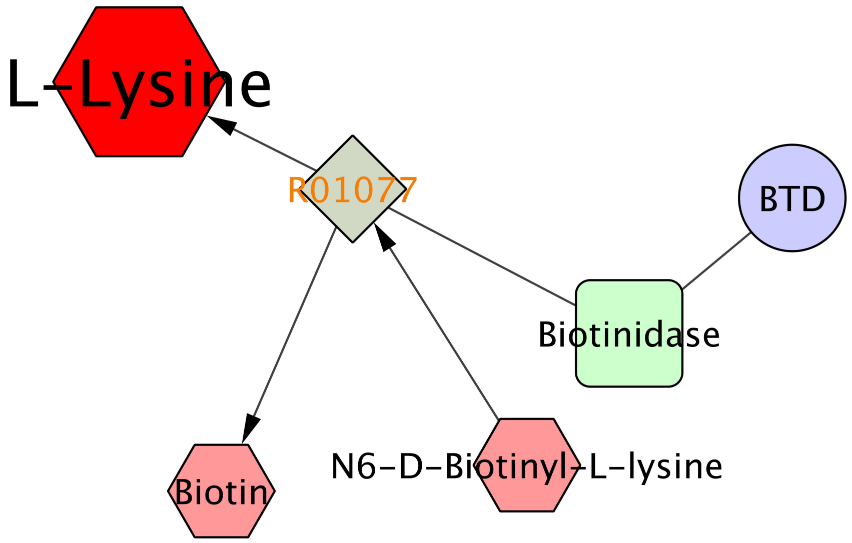

Donkey

Pentose phosphate pathway

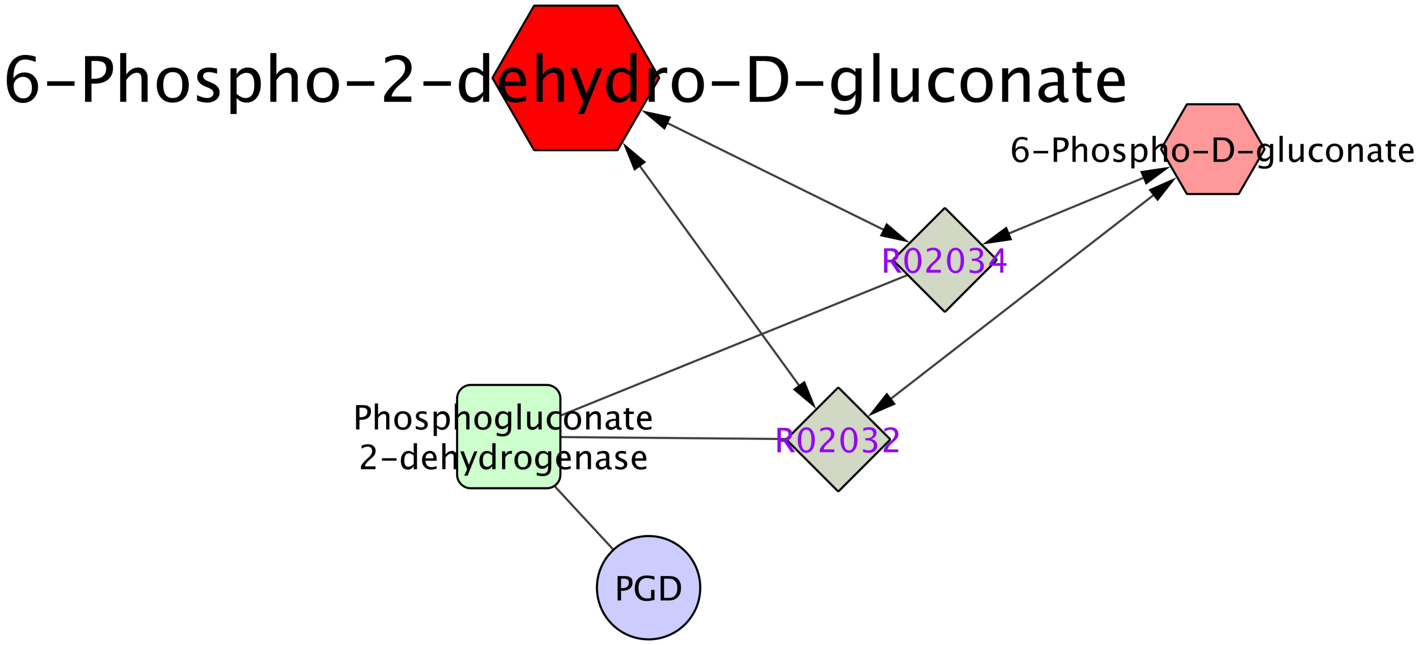

Figure S7

Vitamin H (biotin) metabolism

Cow

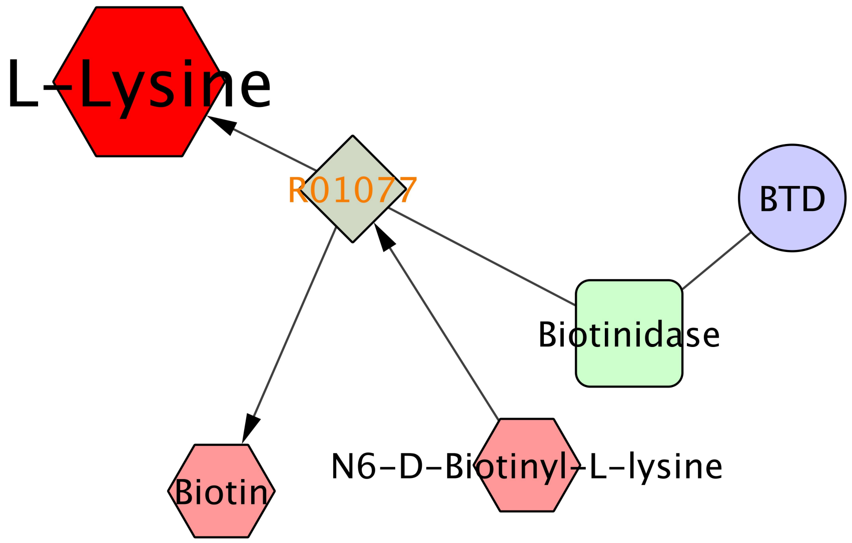

Donkey

Pentose phosphate pathway

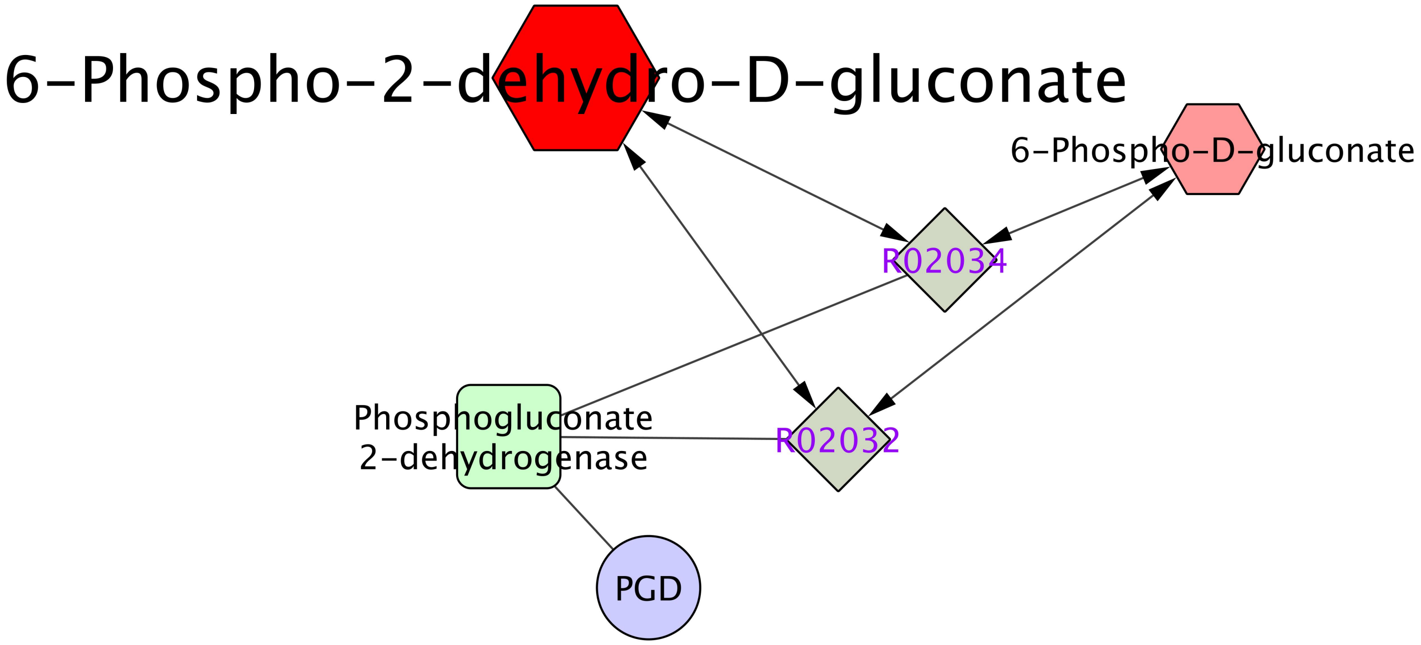

Figure S7

Glycine, serine, alanine and threonine metabolism

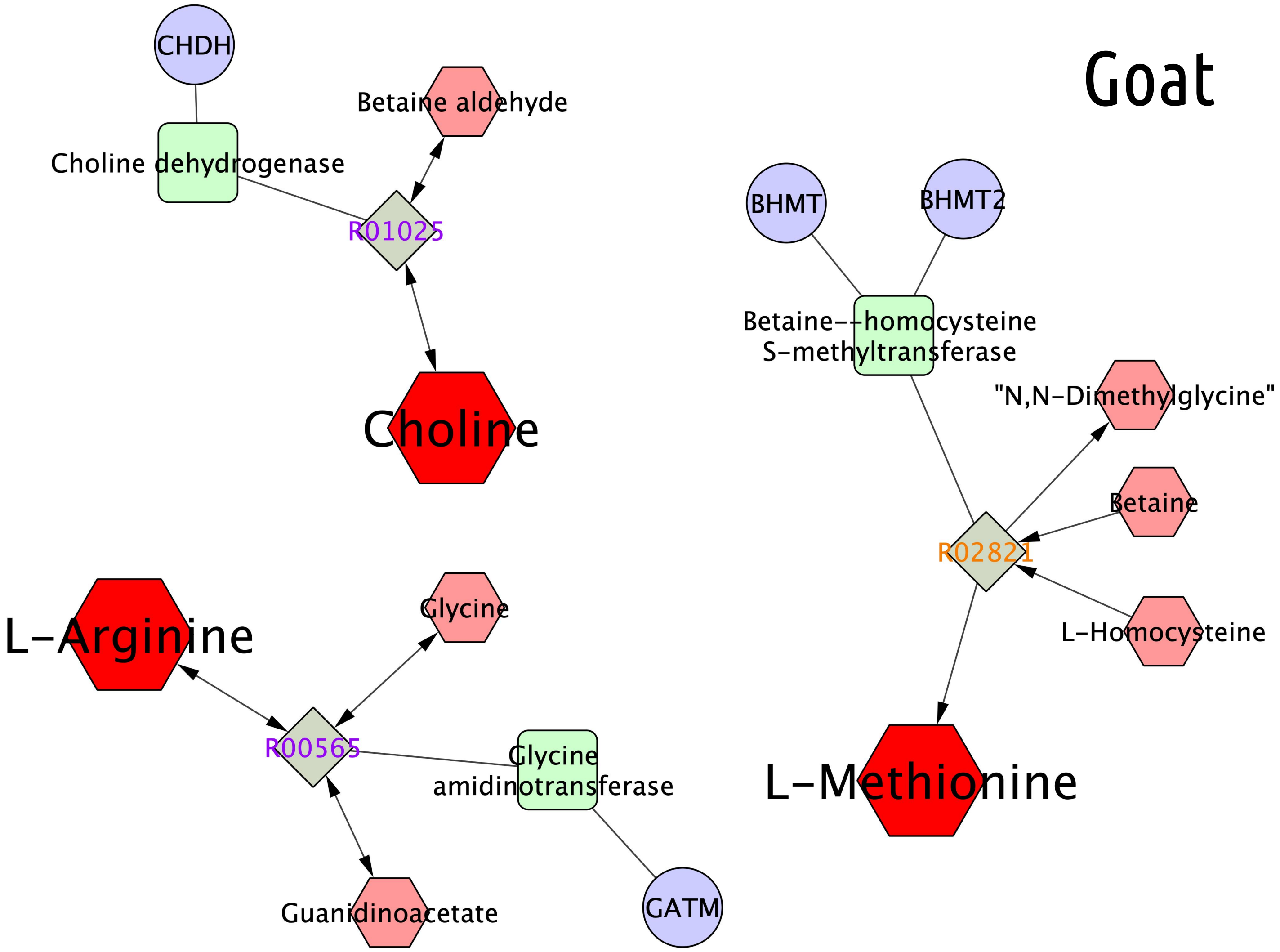

Glycolysis and Gluconeogenesis

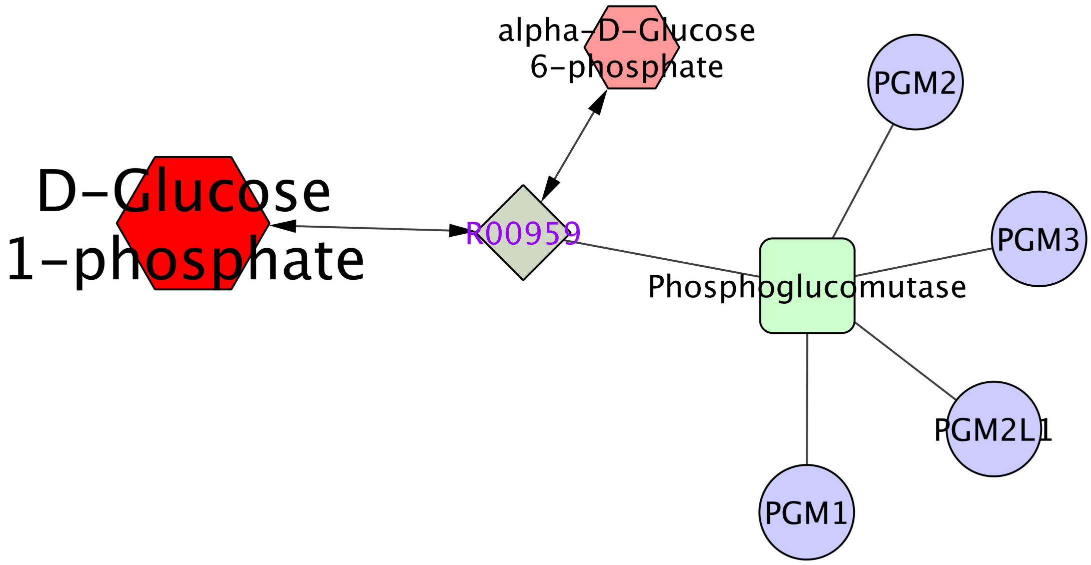

Figure S7

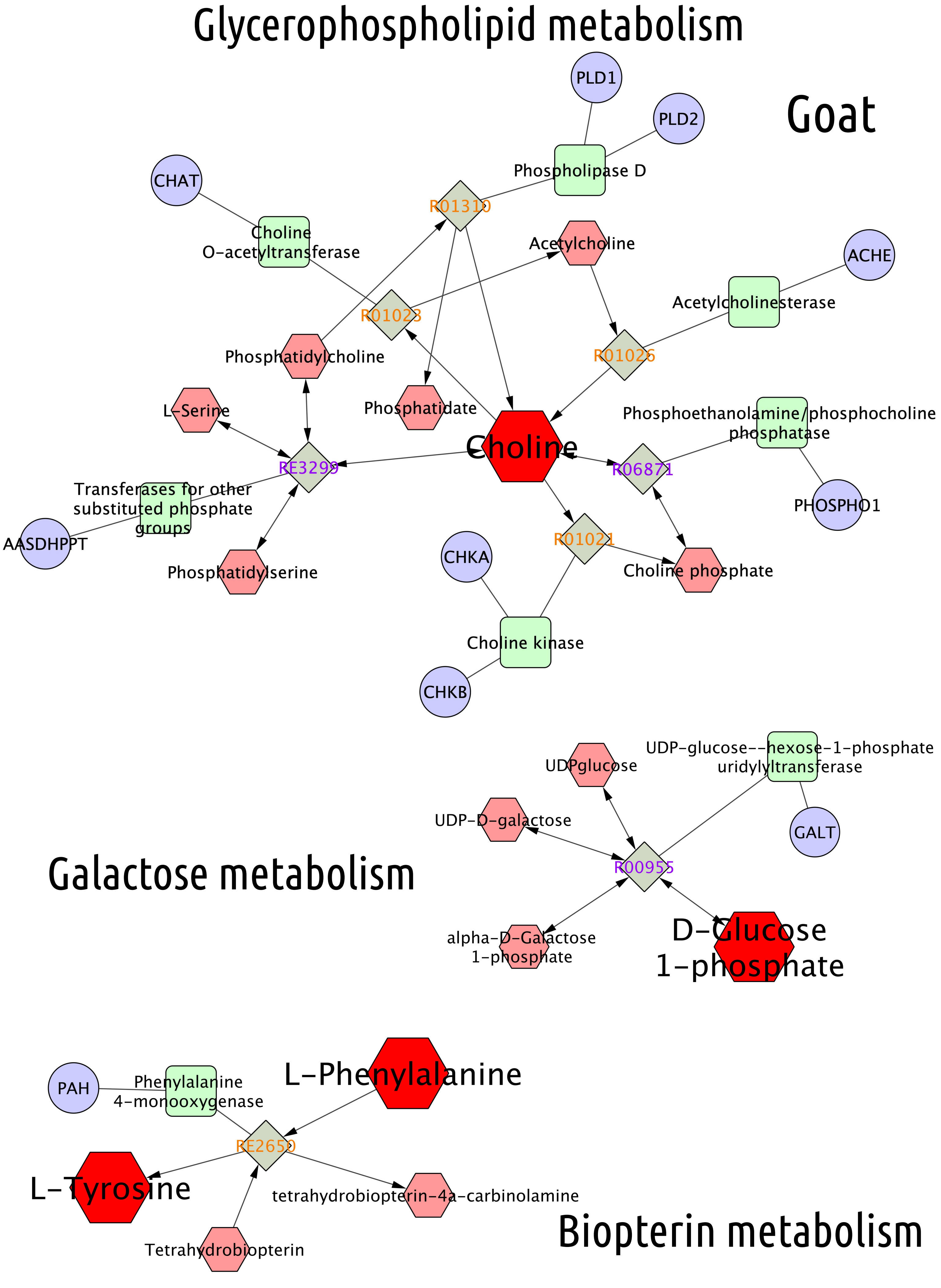

Glycerophospholipid metabolism
Goat
PLD1
PLD2
Phospholipase D
CHAT
R01310
Choline
O-acetyltransferase
Acetylcholine
ACHE
Acetylcholinesterase
R01023
R01026
Phosphatidylcholine
L-Serine
Phosphatidate
Phosphoethanolamine/phosphocholine
phosphatase
Choline
RE3299
R06871
Transferases for other
substituted phosphate
groups
PHOSPHO1
AASDHPPT
R01021
CHKA
Choline phosphate
Phosphatidylserine
Choline kinase
CHKB
UDP-glucose--hexose-1-phosphate
uridylyltransferase
UDPglucose
UDP-D-galactose
GALT
Galactose metabolism
R00955
alpha-D-Galactose
1-phosphate
D-Glucose
1-phosphate
PAH
Phenylalanine
4-monooxygenase
L-Phenylalanine
RE2650
L-Tyrosine
tetrahydrobiopterin-4a-carbinolamine
Tetrahydrobiopterin
Biopterin metabolism

Figure S7
