# Supplementary figures and images for: Anti-Inflammatory Potential of Cow, Donkey and Goat Milk Extracellular Vesicles as Revealed by Metabolomic Profile
Source: Nutrients. 2020 Sep 23;12(10):2908. doi: 10.3390/nu12102908 (PMC7598260; doi:10.3390/nu12102908)

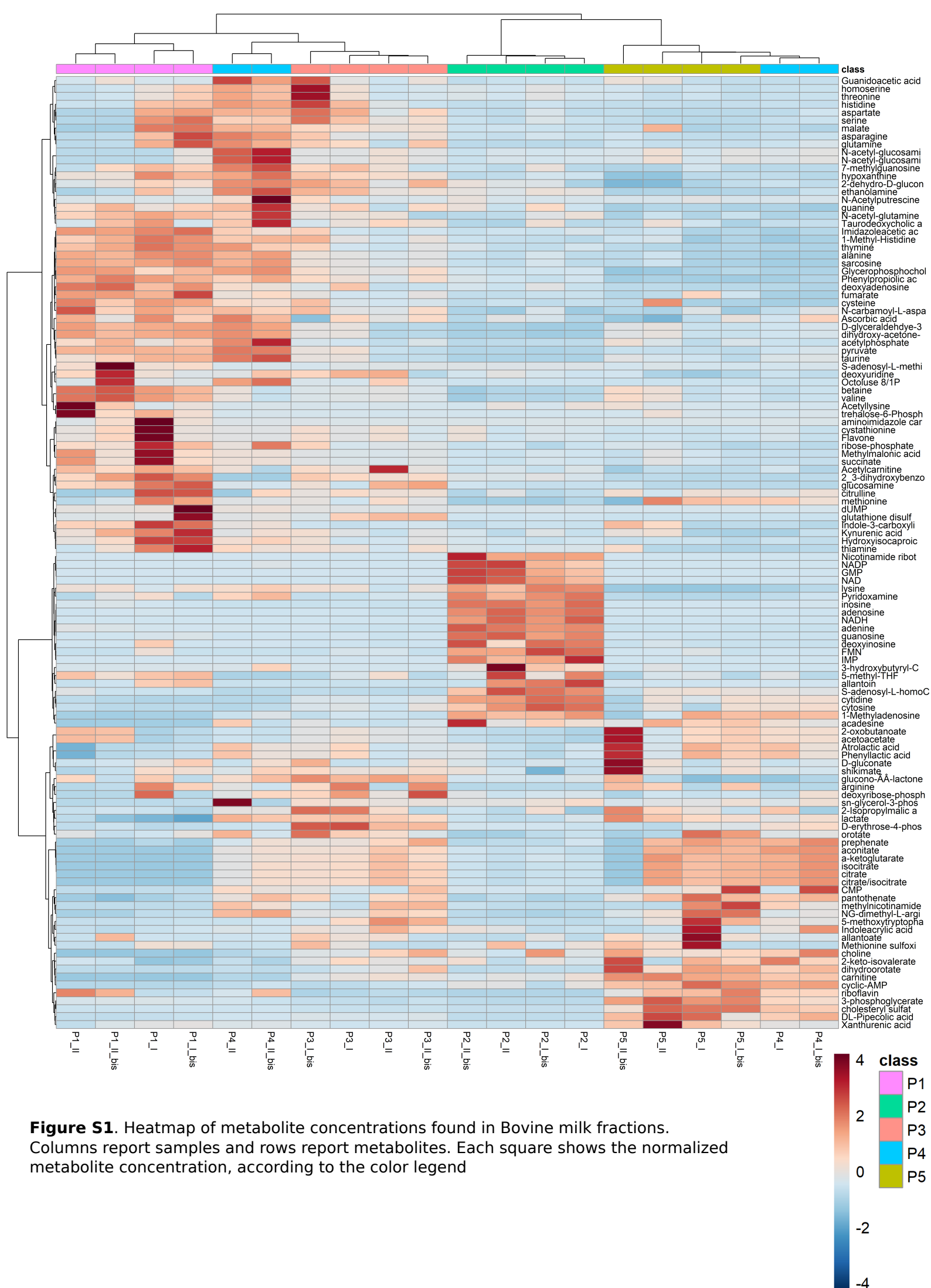

Supplement: Supplementary file 1 [file nutrients-12-02908-s001.zip › Supplementary/FigS1.pdf]

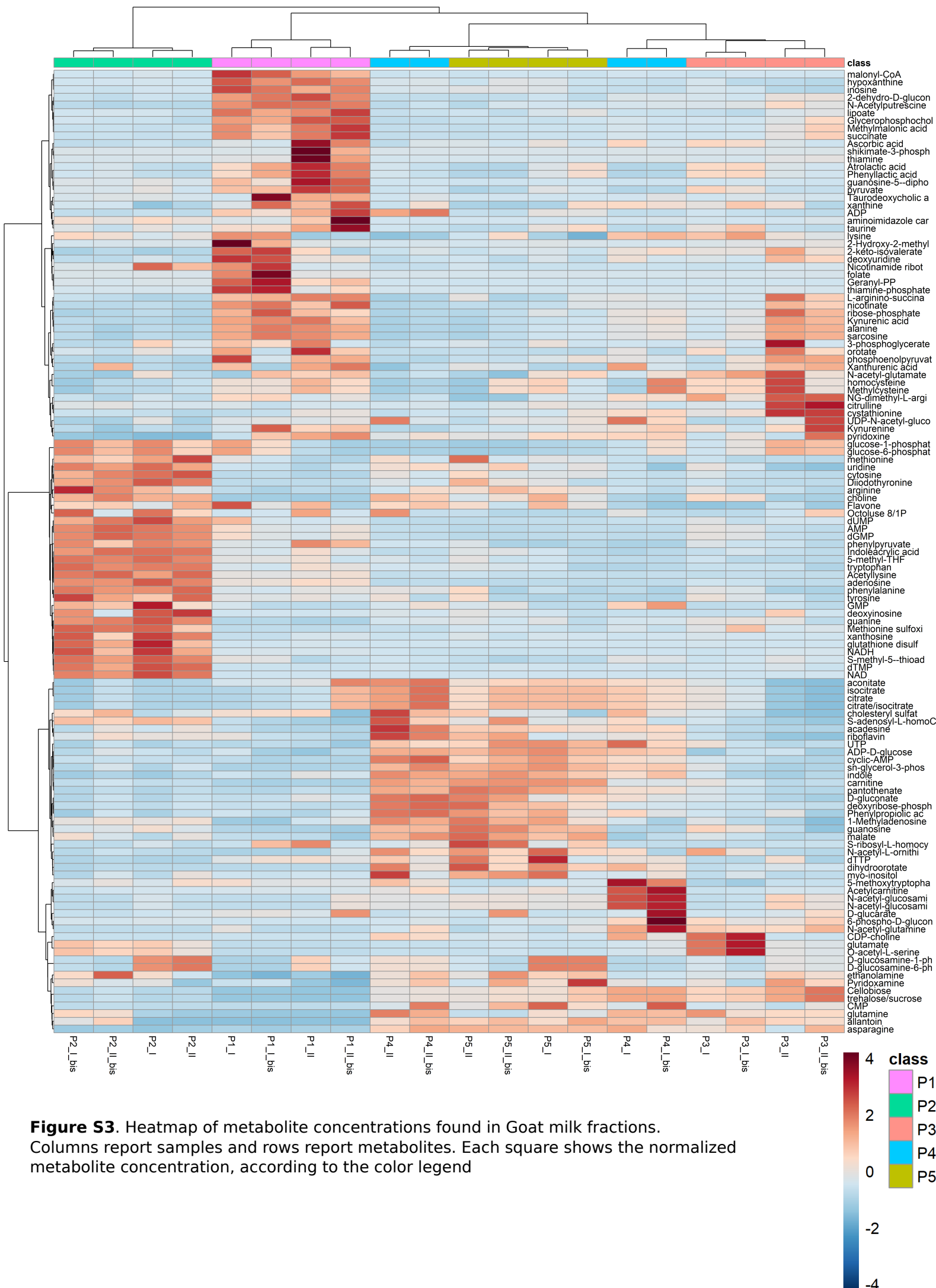

Supplement: Supplementary file 1 [file nutrients-12-02908-s001.zip › Supplementary/FigS3.pdf]
