# Supplementary material for: Anti-Inflammatory Potential of Cow, Donkey and Goat Milk Extracellular Vesicles as Revealed by Metabolomic Profile
Source: Nutrients. 2020 Sep 23;12(10):2908. doi: 10.3390/nu12102908 (PMC7598260; doi:10.3390/nu12102908)
Supplement: Supplementary file 1 [file FigS2.pdf › Supplementary/FigS2.pdf]

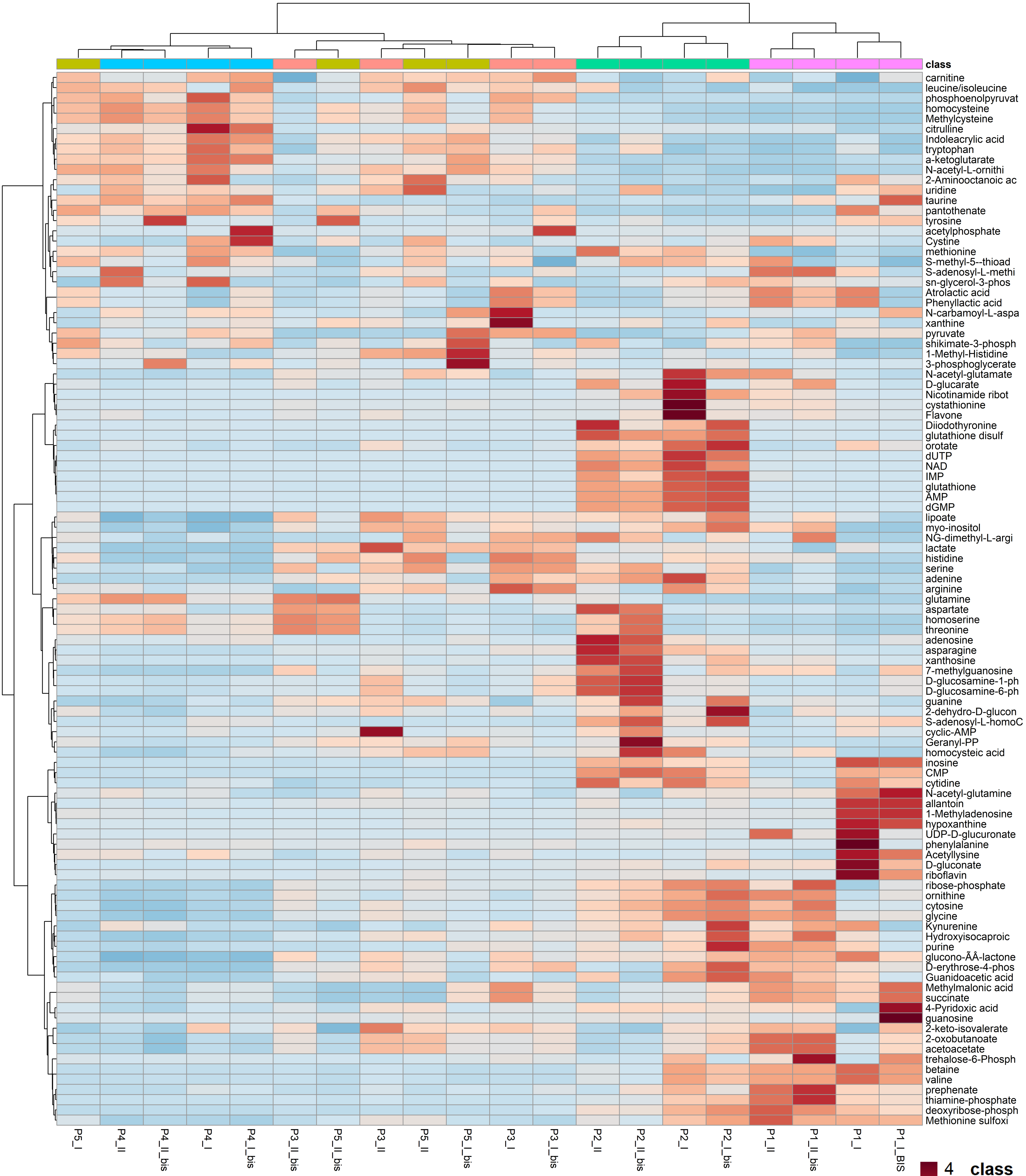

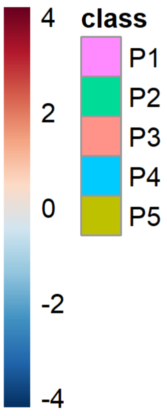

Figure S2. Heatmap of metabolite concentrations found in Donkey milk fractions. Columns report samples and rows report metabolites. Each square shows the normalized metabolite concentration, according to the color legend
